# Crucial role of TFAP2B in the nervous system for regulating NREM sleep

**DOI:** 10.1186/s13041-024-01084-8

**Published:** 2024-02-27

**Authors:** Ayaka Nakai, Mitsuaki Kashiwagi, Tomoyuki Fujiyama, Kanako Iwasaki, Arisa Hirano, Hiromasa Funato, Masashi Yanagisawa, Takeshi Sakurai, Yu Hayashi

**Affiliations:** 1 International Institute for Integrative Sleep Medicine (WPI-IIIS), University of Tsukuba, Ibaraki, 305-8575 Japan; 2 Department of Biological Sciences, Graduate School of Science, University of Tokyo, Tokyo, 113-0033 Japan; 3 Institute of Medicine, University of Tsukuba, Ibaraki, 305-8575 Japan; 4 Department of Anatomy, Toho University Graduate School of Medicine, Tokyo, 143-8540 Japan; 5 Department of Molecular Genetics, University of Texas Southwestern Medical Center, Dallas, TX 75390 USA; 6 Life Science Center for Survival Dynamics, Tsukuba Advanced Research Alliance, University of Tsukuba, Ibaraki, 305-8577 Japan

**Keywords:** Sleep, Mouse, Short-sleeper, TFAP2B

## Abstract

The AP-2 transcription factors are crucial for regulating sleep in both vertebrate and invertebrate animals. In mice, loss of function of the transcription factor AP-2β (TFAP2B) reduces non-rapid eye movement (NREM) sleep. When and where TFAP2B functions, however, is unclear. Here, we used the Cre-loxP system to generate mice in which *Tfap2b* was specifically deleted in the nervous system during development and mice in which neuronal *Tfap2b* was specifically deleted postnatally. Both types of mice exhibited reduced NREM sleep, but the nervous system-specific deletion of *Tfap2b* resulted in more severe sleep phenotypes accompanied by defective light entrainment of the circadian clock and stereotypic jumping behavior. These findings indicate that TFAP2B in postnatal neurons functions at least partly in sleep regulation and imply that TFAP2B also functions either at earlier stages or in additional cell types within the nervous system.

**Supplementary Information:**

The online version contains supplementary material available at 10.1186/s13041-024-01084-8.

## Introduction

Sleep is an evolutionarily conserved state that is crucial for maintaining health in humans [1]. The exact functions and mechanisms of sleep, however, remain elusive. In humans, there are reports of natural short sleepers whose reduced sleep does not lead to daytime fatigue [2]. In cases with an inherited natural short sleep trait, identification and analyses of the causal gene are expected to provide clues to the molecular mechanisms of sleep.

In the present study, we focused on the mouse transcription factor AP-2β (TFAP2B). In humans, partial mutations in *TFAP2B* cause Char syndrome, which is characterized by morphologic abnormalities of the face, limbs, and heart [3, 4]. Two human kindreds with Char syndrome self-reported abnormalities in sleep, namely parasomnia (sleep-walking) and short sleep, respectively [5]. In nematodes, the orthologous transcription factor APTF-1 is critical for specification of the sleep-promoting RIS neuron, and *aptf-1* mutants exhibit severely reduced sleep [6–8]. In fruit flies, knockdown of neuronal *TFAP2* results in reduced nighttime sleep [9]. In mammals, homozygous *Tfap2b*-knock out (KO) mice die shortly after birth [10, 11] whereas heterozygous knockout or partial mutations in mouse *Tfap2b* lead to reduced or fragmented non-rapid eye movement (NREM) sleep [12, 13]. This phenotype is partly explained by the roles of TFAP2B in GABAergic neurons [14], but considering the rather mild effects on NREM sleep of the conditional knockdown (cKD) of *Tfap2b* in GABAergic neurons, the entire picture of the mechanisms by which mouse TFAP2B functions to regulate NREM sleep are unclear. During embryogenesis, *Tfap2b* is expressed in neural crest cells, which generate various cell types, and in the midbrain and hindbrain, where it contributes to the specification of various neurons, including noradrenergic neurons in the locus coeruleus (LC) [15, 16]. Postnatally, *Tfpa2b* is expressed in the paraventricular hypothalamic nucleus (PVH), superior colliculus (SC), parabrachial nucleus (Pb), cerebellum, LC, and nucleus of the solitary tract (NTS), some of which are regions involved in sleep regulation [12]. It is unknown whether TFAP2B acts during development or postnatally in sleep regulation. Here, using the Cre-loxP system, we tested the effect of homozygous deletion of *Tfap2b* in either the nervous system during development or in neurons postnatally.

## Results

### Nervous system-specific* Tfap2b* deletion induces decreased NREM sleep and increased wakefulness

**Fig. 1:**
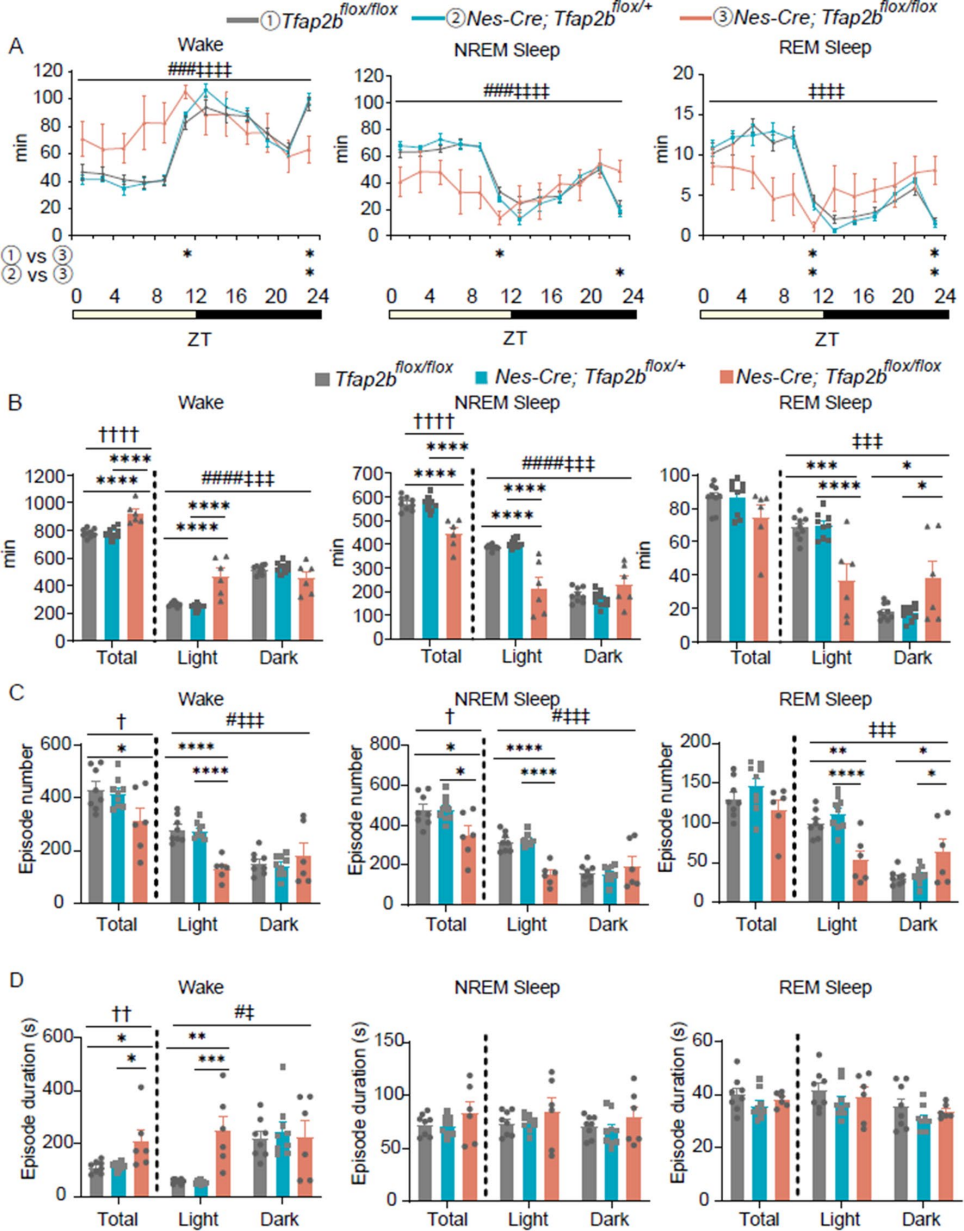
Comparison of the sleep architecture in nervous system-specific *Tfap2b* cKD and control mice. **A** Bi-hourly amount of wakefulness, NREM sleep, and REM sleep across 24 h in male mice. **B** Total amount of wakefulness, NREM sleep, and REM sleep during 24 h, light phase, and dark phase in male mice. *N* = 9 *Tfap2b*^*flox/flox*^ mice (grey), *N* = 9 *Nes-Cre; Tfap2b*^*flox/*+^ mice (blue),* N* = 6 *Nes-Cre; Tfap2b*^*flox/flox*^ mice (pink). **C, D** Episode numbers (**C**) and durations (**D**) of wakefulness, NREM sleep, and REM sleep during 24 h, light phase, and dark phase.* N* = 8 *Tfap2b*^*flox/flox*^ mice (grey), *N* = 9 *Nes-Cre; Tfap2b*^*flox/*+^ mice (blue), *N* = 6 *Nes-Cre; Tfap2b*^*flox/flox*^ mice (pink). ^#^ and ^‡^ indicate a significant main effect of genotype and a significant interaction between genotype and time, respectively, in 2-way repeated-measures ANOVA (^#^, ^‡^*p* < 0.05, ^###^, ^‡‡‡^*p* < 0.001, ^####^, ^‡‡‡‡^*p* < 0.0001). ^†^ indicates significance in the 1-way ANOVA (^†^*p* < 0.05, ^††^*p* < 0.01, ^††††^*p* < 0.0001). * indicates significance in the post-hoc Bonferroni multiple comparison test (*p < 0.05, **p < 0.01, ***p < 0.001, ****p < 0.0001). Data are presented as mean ± SEM

To elucidate whether TFAP2B functions in the nervous system or other tissues in sleep regulation, we used *Nes-Cre* mice to delete *Tfap2b* in a nervous system-specific manner. In *Nes-Cre* mice, Cre recombinase is expressed in neuronal and glial cell precursors of the central and peripheral nervous systems [17]. *Nes-Cre* mice were crossed with *Tfap2b*^*flox/flox*^ mice, in which the *Tfap2b* exon 3 is flanked by loxP. We confirmed that *Tfap2b* exon 3 is deleted in the genomic DNA of the brain in a *Nes-Cre*-dependent manner (Additional file 1: Fig. S1A). Homozygous *Tfap2b* KO mice die shortly after birth [10, 11], whereas the nervous system-specific homozygous *Tfap2b*-conditionally knocked down (cKD) (*Nes-Cre; Tfap2b*^*flox/flox*^) mice lived to adulthood. However, when we crossed *Nes-Cre; Tfap2b*^*flox/*+^ mice with *Tfap2b*^*flox/flox*^ mice to obtain the cKD mice, only 19 out of 176 pups were cKD, which is lower than the expected ratio of 1/4, implying that some of the cKD mice died either prenatally or immediately after birth. The *Nes-Cre; Tfap2b*^*flox/flox*^ male mice had a lower body weight than control male mice (*Tfap2b*^*flox/*+^, *Tfap2b*^*flox/flox*^, or *Nes-Cre; Tfap2b*^*flox/*+^ mice; Additional file 1: Fig. S1B). We compared the sleep architecture between *Nes-Cre; Tfap2b*^*flox/flox*^ and control male mice (Fig. 1). *Nes-Cre; Tfap2b*^*flox/flox*^ mice exhibited increased wakefulness and decreased NREM sleep compared with control mice (Fig. 1B). Decreased NREM sleep could be attributed to a decrease in the episode number (Fig. 1C), whereas the episode duration seemed unaffected (Fig. 1D). In contrast, increased wakefulness could be attributed to a longer episode duration (Fig. 1D), whereas the episode number was rather decreased (Fig. 1C), suggesting that episodes of wakefulness are highly consolidated. When light and dark phases were separately analyzed in *Nes-Cre; Tfap2b*^*flox/flox*^ mice, NREM sleep was largely decreased in the light phase with the amount being comparable between the light and dark phases (Fig. 1B). A similar trend was observed for wakefulness, implying that *Nes-Cre; Tfap2b*^*flox/flox*^ mice lack typical daily fluctuations in the amount of sleep/wake time (Fig. 1B). This trend was even more obvious in rapid eye movement (REM) sleep; while the total amount of REM sleep was unaffected, the amount of REM sleep in the light phase was decreased, whereas that in the dark phase was increased (Fig. 1B). Analyses of the bi-hourly changes in the sleep–wake cycle (Fig. 1A) also revealed a similar trend; the amount of sleep or wakefulness seemed to lack daily oscillations, and when compared with control mice, the amounts of NREM and REM sleep were decreased at some time points in the light phase and increased at some time points in the dark phase, and vice versa for wakefulness (Fig. 1A). To further address whether *Nes-Cre; Tfap2b*^*flox/flox*^ mice exhibit circadian rhythm deficits, we analyzed locomotor activity detected with an infrared sensor. Each individual control mouse exhibited high locomotor activity in the dark phase (Additional file 1: Fig. S2A, B). By contrast, while individual *Nes-Cre; Tfap2b*^*flox/flox*^ mice seemed to have a daily locomotor activity rhythm, the start of the active phase varied among individuals and seemed random (Additional file 1: Fig. S2C), suggesting that the circadian clock is not entrained by external light–dark cycles in these mice. When we defined the active phase and inactive phase based on locomotor activity (see Materials and methods for details), *Nes-Cre; Tfap2b*^*flox/flox*^ mice appeared to have reduced NREM sleep during both the active and inactive periods, whereas REM sleep seemed unaffected (Additional file 1: Fig. S3), providing further support that the sleep deficits in these mice result from a combination of reduced NREM sleep, increased wakefulness, and abnormal light entrainment of the circadian clock. In addition, when the behavior was monitored with a video camera, *Nes-Cre; Tfap2b*^*flox/flox*^ mice frequently displayed stereotypic behaviors such as repeated jumping or climbing (Additional file 2: Video 1). In the electrogram (EEG) power spectrum, *Nes-Cre; Tfap2b*^*flox/flox*^ mice exhibited an increase in the EEG power at approximately 5.5–17 Hz during wakefulness, whereas no obvious defects were detected during sleep (Additional file 1: Fig. S4).

### Postnatal neuron-specific* Tfap2b* deletion induces decreased NREM sleep and increased wakefulness

**Fig. 2:**
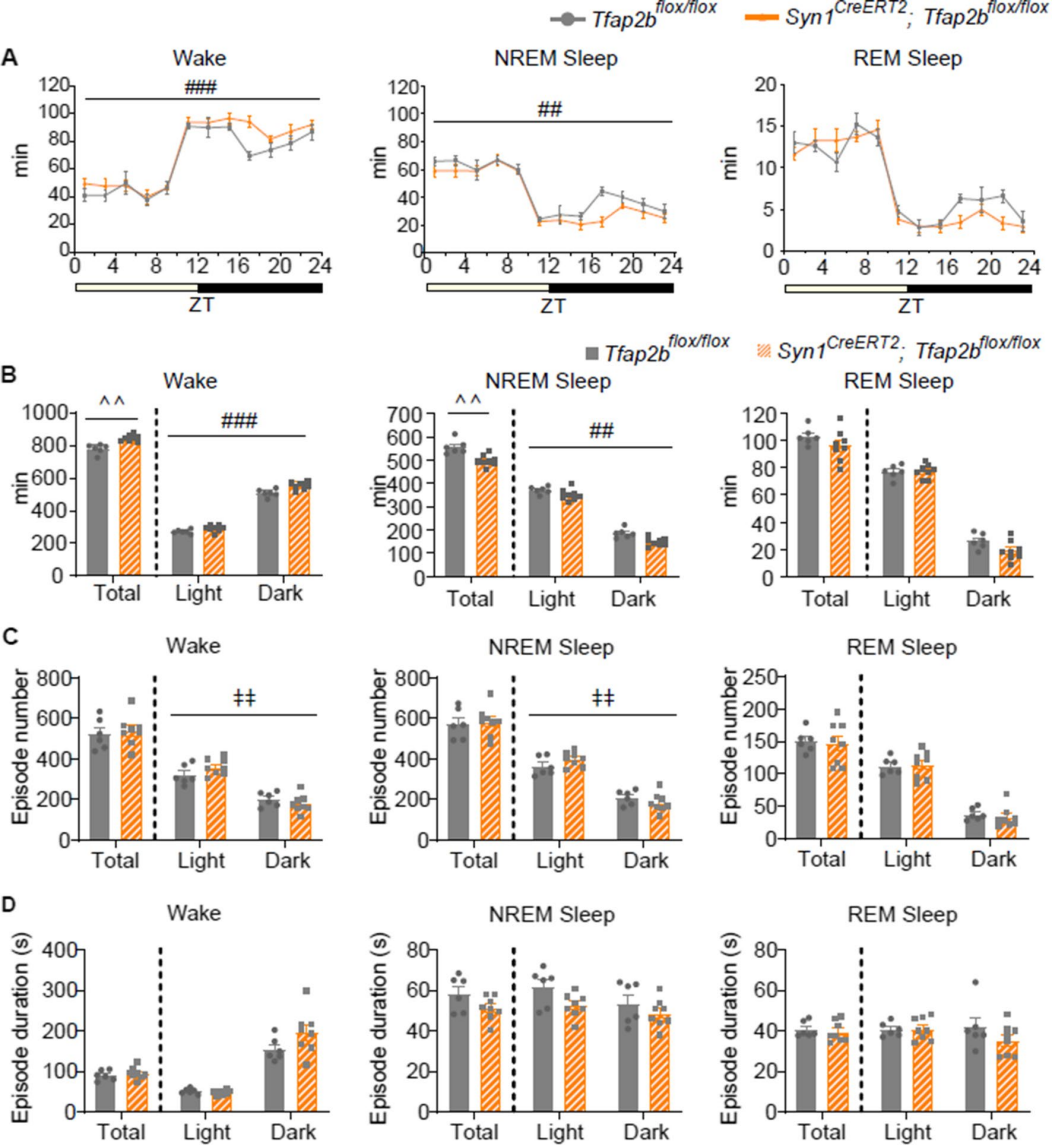
Comparison of the sleep architecture in postnatal neuron-specific *Tfap2b* cKD and control mice. **A** Bi-hourly amount of wakefulness, NREM sleep, and REM sleep across 24 h in tamoxifen-injected (at P14, 17, and 21) male mice. **B** Total amount of wakefulness, NREM sleep, and REM sleep during 24 h, light phase, and dark phase in tamoxifen-injected (at P14, 17, and 21) male mice. **C, D** Episode numbers (**C**) and durations (**D**) of wakefulness, NREM sleep, and REM sleep during 24 h, light phase, and dark phase in tamoxifen-injected (at P14, 17, and 21) male mice. *N* = 6 *Tfap2b*^*flox/flox*^ mice (grey), *N* = 8 *Syn1*^*CreERT2*^*; Tfap2b*^*flox/flox*^ mice (orange). ^#^ and ^‡^ indicate a significant main effect of genotype and significant interaction between genotype and time, respectively, in 2-way repeated-measures ANOVA (^##,‡‡^*p* < 0.01, ^###^*p* < 0.001). ^ indicates significance in the Welch’s test (^^ *p* < 0.01). Data are presented as mean ± SEM

To test whether TFAP2B in neurons acts postnatally in sleep regulation, we crossed *Tfap2b*^*flox/flox*^ mice with *Syn1*^*CreERT2*^ mice, which express CreERT2 specifically in neurons [18]. To delete *Tfap2b* in neurons postnatally, we administered tamoxifen to *Syn1*^*CreERT2*^*; Tfap2b*^*flox/flox*^ mice at postnatal day (P) 14, 17, and 21. We confirmed that *Tfap2b* exon 3 is deleted in the genomic DNA of the brain in a *Syn1*^*CreERT2*^-dependent manner (Additional file 1: Fig. S5A). These *Syn1*^*CreERT2*^*; Tfap2b*^*flox/flox*^ mice survived to adulthood and exhibited no significant difference in body weight (Additional file 1: Fig. S5B). Repetitive jumping or climbing behaviors were not observed (Additional file 3: Video 2). We compared the sleep architecture between *Syn1*^*CreERT2*^*; Tfap2b*^*flox/flox*^ and *Tfap2b*^*flox/flox*^ male mice administered with tamoxifen (at P14, 17, and 21) (Fig. 2). *Syn1*^*CreERT2*^*; Tfap2b*^*flox/flox*^ mice showed increased wakefulness and decreased NREM sleep (Fig. 2A, B), similar to *Nes-Cre; Tfap2b*^*flox/flox*^ mice, although the phenotype seemed milder. Perhaps due to the mild phenotype, episode numbers and durations were not significantly affected (Fig. 2C, D). When light and dark phases were analyzed separately, the amount of wakefulness and NREM sleep seemed to be affected throughout both phases (Fig. 2B). Unlike *Nes-Cre; Tfap2b*^*flox/flox*^ mice, the circadian rhythm seemed normal, with a high amount of wakefulness during the dark phase and a low amount during the light phase, and vice versa for NREM and REM sleep (Fig. 2A, B). EEG power spectra during wakefulness, NREM sleep, or REM sleep appeared unaffected (Additional file 1: Fig. S6). We also tested whether sleep homeostasis is normal in these mice. Mice were subjected to 5 h of sleep deprivation (SD) at the beginning of the light phase. Both *Syn1*^*CreERT2*^*; Tfap2b*^*flox/flox*^ mice and *Tfap2b*^*flox/flox*^ mice showed an increase in NREM sleep during the following 7 h of the light phase after SD (Additional file 1: Fig. S7A, B). Sleep architecture and EEG power spectra were also similar (Additional file 1: Fig. S7C-E), suggesting that the *Syn1*^*CreERT2*^*; Tfap2b*^*flox/flox*^ mice exhibit a normal response to SD. Importantly, a previous study showed that the *Syn1*^*CreERT2*^ allele does not affect the amount of wakefulness, NREM sleep, and REM sleep [18]. However, we cannot rule out the possibility that it might have had some effect on the sleep architecture or the EEG power spectra, although these factors seemed not to differ between *Syn1*^*CreERT2*^*; Tfap2b*^*flox/flox*^ and *Tfap2b*^*flox/flox*^ mice.

### Differential patterns of recombination in *Nes-Cre* and *Syn1*^*CreERT2*^ mice.

**Fig. 3:**
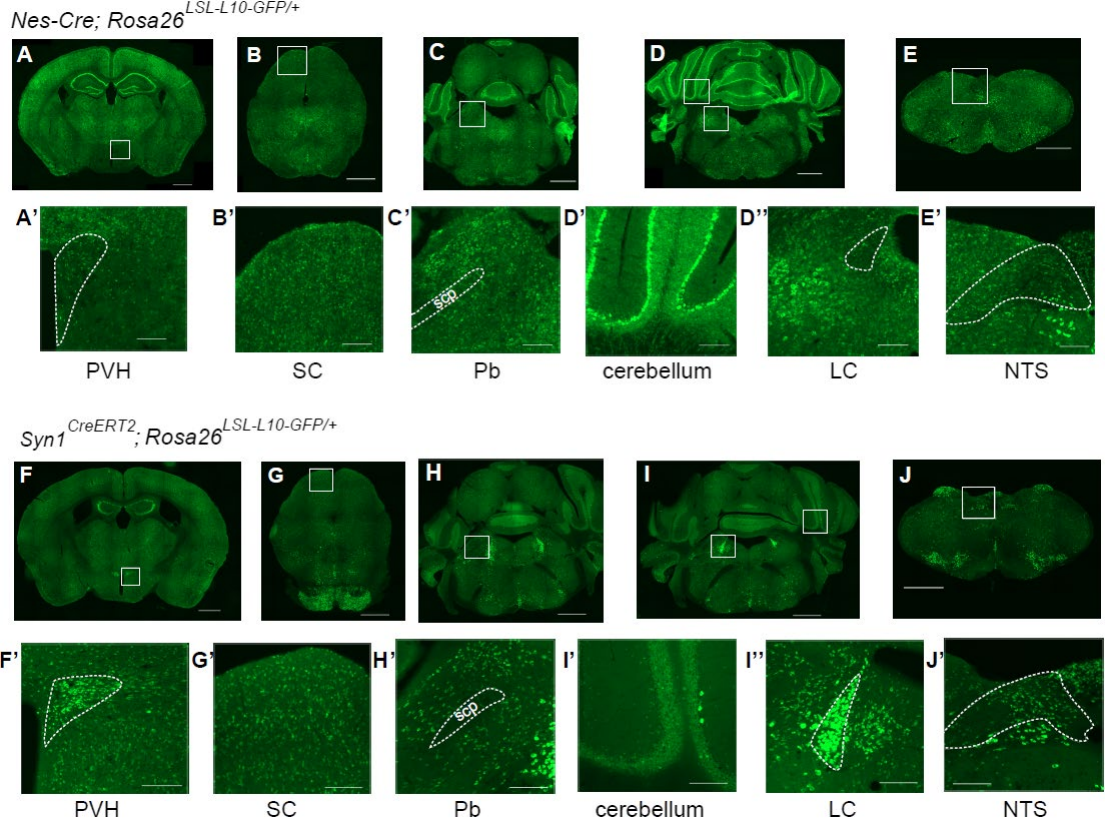
Comparison of the Cre-mediated recombination patterns in *Nes-Cre* and *Syn1*^*CreERT2*^ mice. (**A**–**J**’) Images of areas where GFP signals were detected in *Nes-Cre; Rosa26*^*LSL−L10−GFP/*+^ mice (**A–E’**) and tamoxifen-injected (at P14, 17, and 21) *Syn1*^*CreERT2*^*; Rosa26*^*LSL−L10−GFP/*+^ mice (**F–J’**). PVH, paraventricular hypothalamic nucleus (**A’, F’**); SC, superior colliculus (**B’, G’**); Pb, parabrachial nucleus (**C’, H’**); cerebellum (**D’, I’**), LC, locus coeruleus (**D’’, I’’**); NTS, solitary tract nucleus (**E’, J’**), scp, superior cerebellar peduncle. Scale bars in (**A**–**J**), 1 mm; scale bars in (**A**’–**J**’), 200 µm. Representative images from a replicate of *N* = 2 *Nes-Cre; Rosa26*^*LSL−L10−GFP/*+^ mice, *N* = 4 *Syn1*^*CreERT2*^*; Rosa26*^*LSL−L10−GFP/*+^ mice are shown

To reveal the Cre-mediated recombination patterns in *Nes-Cre* and *Syn1-CreERT2* mice, we crossed these mice with *Rosa26*^*LSL-L10-GFP*^ Cre-reporter mice. In the mature brain, TFAP2B is expressed in the PVH, SC, Pb, cerebellum, LC, and NTS [12]. Within these areas, *Nes-Cre; Rosa26*^*LSL-L10-GFP/*+^ mice had dense GFP signals in the SC, Pb, cerebellum, and NTS, whereas GFP signals were sparse in the PVH and almost undetectable in the LC (Fig. 3A–E’). *Syn1*^*CreERT2*^*; Rosa26*^*LSL-L10-GFP/*+^ mice injected with tamoxifen (at P14, 17, and 21) had dense GFP signals in the PVH, SC, Pb, LC, and NTS, whereas GFP signals were sparse in the cerebellum (Fig. 3F–J’). GFP signals in the LC mostly overlapped with tyrosine hydroxylase, suggesting that the recombination occurred in noradrenergic neurons (Additional file 1: Fig. S8).

## Discussion

Heterozygous deletion of *Tfap2b* leads to reduced NREM sleep [12]. The present study showed that homozygous deletion of *Tfap2b* in the nervous system during development leads to a large reduction in NREM sleep. Moreover, homozygous deletion of *Tfap2b* in neurons postnatally reduced NREM sleep, although to a milder extent. These findings indicate that TFAP2B in postnatal neurons at least partly functions in sleep regulation and imply that TFAP2B also functions either at earlier stages or in additional cell types.

Consistent with previous studies [19], in *Nes-Cre* mice, Cre recombination was not detected in various brain regions. Within areas that postnatally express *Tfap2b*, Cre recombination was detected in the SC in both *Nes-Cre* and *Syn1*^*CreERT2*^ mice. GABAergic neurons in the SC regulate sleep by inhibiting dopaminergic neurons in the ventral tegmental area [20], and loss of function in these neurons might be partly responsible for the reduced NREM sleep in both *Syn1*^*CreERT2*^*; Tfap2b*^*flox/flox*^ and *Nes-Cre; Tfap2b*^*flox/flox*^ mice. This is consistent with a recent study demonstrating that deletion of *Tfap2b* specifically in GABAergic neurons leads to reduced NREM sleep [14]. The effect of deleting *Tfap2b* in GABAergic neurons is limited, however, suggesting that TFAP2B in other neuronal types also functions in sleep regulation. Among areas that postnatally express *Tfap2b*, Cre recombination was also detected in the NTS. Glutamatergic neurons in the NTS promote NREM sleep [21], and loss of function in these neurons might also contribute to reduce NREM sleep. By contrast, among areas that postnatally express *Tfap2b*, Cre recombination was almost undetectable in the LC in *Nes-Cre* mice. TFAP2B is required for the synthesis of noradrenaline in the LC [22], which in turn is crucial for maintaining wakefulness [23]. Thus, the milder reduction of NREM sleep in *Syn1*^*CreERT2*^*; Tfap2b*^*flox/flox*^ mice compared with *Nes-Cre; Tfap2b*^*flox/flox*^ mice might be partly due to the additional functional loss of LC noradrenergic neurons in the *Syn1*^*CreERT2*^*; Tfap2b*^*flox/flox*^ mice, which might have counteracted the NREM sleep-reducing effect of deleting *Tfap2b*. Another possibility might be that TFAP2B also functions in glial cells or neural progenitor cells to regulate sleep and that deletion of *Tfap2b* in these cells are responsible for the more severe sleep phenotypes of *Nes-Cre; Tfap2b*^*flox/flox*^ mice compared with *Syn1*^*CreERT2*^*; Tfap2b*^*flox/flox*^ mice. Indeed, homozygous *Tfap2b* KO affects expression of genes involved in glial cell differentiation [14]. These possibilities should be tested in the future using mice that express Cre specifically in either neurons or glial cells.

*Nes-Cre; Tfap2b*^*flox/flox*^ mice had prolonged wake episodes, during which they exhibited stereotypic behaviors, including repeated jumping and climbing. Similar stereotypic behaviors are observed in mice with knockout of dopamine transporter 1 (*Dat1*) and those with knockout of SH3 and multiple ankyrin repeat domains 2 (*Shank2*) [24, 25] and are thought to share properties with human stereotypic behaviors observed in various psychiatric or developmental disorders [26]. This phenotype is likely due to loss of *Tfap2b* either during the fetal period or in glial cells as such abnormal behavior was not observed in *Syn1*^*CreERT2*^*; Tfap2b*^*flox/flox*^ mice.

In addition, *Nes-Cre; Tfap2b*^*flox/flox*^ mice exhibited a deficit in light entrainment of the circadian rhythm. Intrinsically photosensitive retinal ganglion cells (ipRGCs) function as photosensors for this light entrainment [27]. Within the retina, TFAP2B is crucial for the development of horizontal and amacrine cells [28, 29], and considering the deficits in light entrainment, TFAP2B might also have a role in the development of ipRGCs.

Whereas *Syn1*^*CreERT2*^*; Tfap2b*^*flox/flox*^ mice showed reduced basal NREM sleep, they responded normally to SD with increased amounts of NREM sleep in the subsequent hours. Thus, TFAP2B might not be required in postnatal neurons for the homeostatic regulation of sleep, but TFAP2B in other cell types may still play a role. We could not conduct a similar SD experiment with *Nes-Cre; Tfap2b*^*flox/flox*^ mice because the beginning of the inactive phase varied among individual mice.

This study showed that postnatal neurons require TFAP2B to regulate sleep. To further narrow down the brain regions and neuronal subtypes in which *Tfap2b* is involved in regulating sleep, additional studies using region-specific Cre-driver lines or local microinjection of adeno-associated viral vectors carrying Cre are required. Once the responsible populations of neurons are identified, further studies using single-cell RNA sequencing may identify the target genes of TFAP2B that are required for regulating NREM sleep and contribute to a deeper understanding of the molecular mechanisms of sleep.

## Materials and methods

### Mice

All animal experiments were approved by the institutional animal care and use committee of the University of Tsukuba. All animals were maintained according to the institutional guidelines of the animal facilities of the Laboratory of Animal Resource Center, University of Tsukuba. Mice were maintained and bred within the International Institute for Integrative Sleep Medicine under a 12-h light/dark cycle and with free access to food and water. Male *Nes-Cre; Tfap2b*^*flox/flox*^, *Syn1*^*CreERT2*^; *Tfap2b*^*flox/flox*^, and corresponding control mice aged 10–13 weeks were used for sleep analyses. To obtain Tfap2b cKD mice, *Tfap2b*^*tm1a(EUCOMM)Wtsi/+*^ mice were crossed with mice carrying the *CAG-flp* allele [30] to remove a genomic DNA fragment containing IRES:lacZ and a neomycin-resistant cassette flanked by FRT. Homozygous floxed *Tfap2b* exon 3 (*Tfap2b*^*flox/flox*^) mice were mated to mice carrying the *Nes-Cre* transgene [17] or the *Syn1*^*CreERT2*^ allele [18] to induce Cre-mediated recombination in a nervous system-specific manner or a neuron-specific manner, respectively. 

 To confirm the recombination sites in the *Nes-Cre* and *Syn1*^*CreERT2*^ mice, *Nes-Cre* and *Syn1*^*CreERT2*^ mice were mated with the GFP reporter strain *B6.129S4-Gt (ROSA)26Sor*^*tm1(CAG-EGFP/Rpl10a-birA) Wtp/J*^ (*Rosa26*^*LSL-L10-GFP*^, Jackson Laboratory ; #022367) [31].

To induce CreERT2-mediated recombination, 500 μg of tamoxifen (Toronto Research Chemicals Inc.; T006000) dissolved in corn oil was administered intraperitoneally at P14, P17, and P21.

### Genotyping

Genotyping of mice carrying *Tfap2b*^*flox*^, *Nes-Cre*, *Syn1*^*CreERT2*^, or *Rosa26*^*LSL-L10-GFP*^ was performed using the following primers. *Tfap2b*^*flox*^: 5′-AAGGCGCATAACGATACCAC-3′, 5′-CCGCCTACTGCGACTATAGAGA-3′, 5′-GACATCCTACAATGCACAGCT-3′, and 5′- TTGCTGTGAGCTAAGAGCTTC-3′ (*Tfap2b*^*flox*^: 218 bp, *Tfap2b*^*+*^: 381 bp), *Nes-Cre*: 5′-GACGATGCAACGAGTGATGA-3′ and 5′-AGCATTGCTGTCACTTGGTC-3′ (*Nes-Cre*: 300 bp), *Syn1*^*CreERT2*^: 5′-TGCCTCCACCTTGTCTCTCT-3′, 5′-AACAAAGGCATGGAGCATCT-3′, and 5′-GATCTGGAGGTGACCAGGAA-3′ (*Syn1*^*CreERT2*^: 443 bp, *Syn1*^*+*^: 387 bp), *Rosa26*^*LSL-L10-GFP*^: 5′-AAGGGAGCTGCAGTGGAGTA-3′, 5′-CCGAAAATCTGTGGGAAGTC-3′, 5′-AAGATCCGCCACAACATCG-3′, and 5′-TTCTCGTTGGGGTCTTTGCT-3′ (*Rosa26*^*LSL-L10-GFP*^: 146 bp, *Rosa26*^*+*^: 297 bp). 

### PCR using genomic DNA extracted from brain 

 Each brain was harvested and divided in half at the midline and frozen with liquid nitrogen. Genomic DNA was extracted using ISOSPIN Tissue DNA Kit (NIPPON GENE; 316-08891). PCR was performed using the following primers. *Tfap2b*^*−*^: 5′-AAGGCGCATAACGATACCAC-3′ and 5′-ACTGATGGCGAGCTCAGACC-3′ (*Tfap2b*^*−*^: 174 bp, *Tfap2b*^*flox*^: 1024 bp), *Chn1*: 5′-AGGGCTTTCCTTGCTGTGTC-3′ and 5′-TAGGTCCCTTCTCATGAACC-3′ (*Chn1*^*+*^: 120 bp).

### EEG/ electromyogram (EMG) and locomotor recording

EEG and EMG signals were recorded from freely moving mice at the age of 10–13 weeks according to the method described in a previous study [32], with some modifications. First, 8–10 weeks-old mice were implanted with EEG/EMG electrodes. Stainless steel EEG electrodes were implanted epidurally over the cerebellum and parietal cortex, and EMG electrodes were embedded into the trapezius muscles bilaterally. Mice were allowed to recover from the surgery in their home cage for at least 4 days. Then, mice were placed in a sleep recording chamber and habituated for at least 5 days. Subsequently, EEG/EMG signals were recorded from the onset of the light phase. EEG/EMG signals were filtered (bandpass 0.5–250 Hz), collected, and digitized at a 512 Hz sampling rate using VitalRecorder (Kissei Comtec). Video and infrared signals were also recorded from the start of the light phase. Locomotor activity detected by infrared signals was used to determine the onset of the active phase, which was automatically done using a template-matching algorithm in the ClockLab software. Briefly, the onset of the active phase was defined as the start point of 5 consecutive hours of high locomotor activity (above the 20% percentile of average locomotor activity) that followed 5 consecutive hours of low locomotor activity. 12 consecutive hours from the onset of the active phase were categorized as the active phase, and the remaining 12 consecutive hours were categorized as the inactive phase.

### EEG/EMG analysis

EEG/EMG data were divided into 4-s epochs (time window), and EEG data were further subjected to fast Fourier transform analyses using SleepSign (Kissei Comtec). The vigilance state in each epoch was manually classified as REM sleep, NREM sleep, or wake based on absolute delta (0.5–4 Hz) power, theta (6–10 Hz) power to delta power ratio, and the integral of EMG signals. If a single epoch contained multiple states, the state with the highest occupancy was assigned. The EEG power spectrum of each state was calculated and normalized by EEG power at 16–30 Hz averaged across 24 h. Epochs that contained multiple stages or presumable large movement-derived artifacts in the EEG data were included in the stage analysis but excluded from the EEG power spectrum analysis. 

### Sleep deprivation (SD)

SD was performed for 5 h during ZT0-ZT5. We monitored EEG/EMG to detect sleep. The cage was changed at the start of SD. Whenever sleep was detected, Kimwipes or marbles were placed in or removed from the cage, or the cage was gently tapped. 

### Histology

Deeply anesthetized mice were killed by injecting a lethal dose of anesthetic and transcardially perfused with 0.1 M phosphate-buffered saline (PBS) followed by 4% paraformaldehyde (w/v) in 0.1 M PBS or 10% formalin neutral buffer solution. The brains were postfixed in the same fixative for 1 day and subsequently equilibrated in 30% sucrose (w/v) in PBS. The brains were sectioned at 40 μm using a sliding microtome (Yamato Kohki) or a cryostat (Leica).

### Immunohistochemistry

Sections were washed 3 times in tris-buffered saline with Tween20 (TBST; 50 mM Tris-HCl, pH 7.6, 150 mM NaCl, 0.05% Tween20), once in 0.3% H_2_O_2_/TBST, and 3 times again in TBST. The sections were then blocked for 30 min in blocking buffer [tris-buffered saline with 0.5% Blocking Reagent (Perkin Elmer; FP1012)]. The sections were incubated with a primary antibody [1/2000 mouse anti-tyrosine hydroxylase (Sigma; T2928)]. After washing 3 times in TBST, the sections were incubated with a secondary antibody [1/500 horseradish peroxidase-conjugated donkey anti-mouse IgG (Abcam; ab7061)] for 2 h. After washing 3 times in TBST, the sections were incubated with Tyramide Signal Amplification (TSA) plus Cyanine 5 reagent (Perkin Elmer; NEL745001KT) for 30 min. Finally, after washing 4 times in TBST, the sections were mounted on a slide glass with mounting medium (ThermoFisher; TA-006-FM). Images were captured with a digital slide scanner (Carl Zeiss; AxioScan.Z1) or a confocal microscope (Carl Zeiss; LMS800). 

### Quantification and statistical analysis 

The experimenter was blinded to the genotype during sleep scoring. All statistical analyses were performed using Prism9 (GraphPad), and statistical significance was set at *p* < 0.05. Where applicable, all statistical tests were 2-tailed.

### Supplementary Information

**Additional file 1. Fig. S1.**  Confirmation of *Tfap2b* deletion and comparison of bodyweight in nervous system-specific *Tfap2b* cKD and control mice. **Fig. S2.** Locomotor activity of nervous system-specific *Tfap2b* cKD mice. **Fig. S3.** Comparison of the amount of sleep/wake in nervous system-specific *Tfap2b* cKD and control mice during the active and inactive phase determined by locomotor activity.  **Fig. S4.** Comparison of EEG power spectra in nervous system-specific *Tfap2b* cKD and control mice. **Fig. S5.** Confirmation of *Tfap2b* deletion and comparison of bodyweight in postnatal neuron-specific *Tfap2b* cKD and control mice. **Fig. S6.** Comparison of EEG power spectra in postnatal neuron-specific *Tfap2b* cKD and control mice. **Fig. S7.** Comparison of the sleep architecture after sleep deprivation (SD) in postnatal neuron-specific *Tfap2b* cKD and control mice. **Fig. S8.** Analyses of the Cre-mediated recombination pattern in *Syn1*^*CreERT2*^ mice at the LC.

**Additional file 2. Video 1. **Representative video of nervous system-specific *Tfap2b* cKD and control mice. (A-D) Video recordings of an individual *Nes-Cre; Tfap2b*^*flox/+*^ (A), *Tfap2b*^*flox/flox*^ (B), *Nes-Cre; Tfap2b*^*flox/+*^ (C), or *Nes-Cre; Tfap2b*^*flox/flox*^ (D) male mouse during the light phase.

**Additional file 3: Video 2.** Representative video of postnatal neuron-specific *Tfap2b* cKD and control mice. (A, B) Video recordings of an individual *Syn1*^*CreERT2*^; *Tfap2b*^*flox/flox*^ (A) or *Tfap2b*^*flox/flox*^ (B) male mouse injected with tamoxifen (at P14, 17, and 21) during the light phase.

## Data Availability

All data are available upon reasonable request to the corresponding author.

## Abbreviations

REM sleep: Rapid eye movement sleep
NREM sleep: Non-rapid eye movement sleep
EEG: Electroencephalogram
EMG: Electromyogram
GFP: Green fluorescent protein
TFAP2B: Transcription factor AP-2β
KO: Knocked out
cKD: Conditional knockdown
WT: Wild-type
NES: Nestin
SYN1: Synapsin 1
CHN1: Chimerin 1
PVH: Paraventricular hypothalamic nucleus
SC: Superior colliculus
Pb: Parabrachial nucleus
LC: Locus coeruleus
NTS: Nucleus of the solitary tract
SD: Sleep deprivation
TH: Tyrosine hydroxylase
ipRGCs: Intrinsically photosensitive retinal ganglion cells
SEM: Standard error of the mean

## References

[1] Miyazaki S, Liu C-Y, Hayashi Y (2017). Sleep in vertebrate and invertebrate animals, and insights into the function and evolution of sleep. Neurosci Res.

[2] Shi G, Wu D, Ptáček LJ, Fu YH. Human genetics and sleep behavior. Curr Opin Neurobiol. 2017; 43–9. 10.1016/j.conb.2017.02.015 PMC5511083 28325617

[3] Davidson HR (1993). A large family with patent ductus arteriosus and unusual face. J Med Genet.

[4] Satoda M, Pierpont MEM, Diaz GA, Bornemeier RA, Gelb BD (1999). Char syndrome, an inherited disorder with patent ductus arteriosus, maps to chromosome 6p12-p21. Circulation.

[5] Mani A, Radhakrishnan J, Farhi A, Carew KS, Warnes CA, Nelson-Williams C (2005). Syndromic patent ductus arteriosus: evidence for haploinsufficient TFAP2B mutations and identification of a linked sleep disorder. Proc Natl Acad Sci.

[6] Konietzka J, Fritz M, Spiri S, McWhirter R, Leha A, Palumbos S (2020). Epidermal growth factor signaling promotes sleep through a combined series and parallel neural circuit. Curr Biol.

[7] Turek M, Lewandrowski I, Bringmann H (2013). An AP2 transcription factor is required for a sleep-active neuron to induce sleep-like quiescence in *C. elegans*. Curr Biol.

[8] Turek M, Besseling J, Spies J-P, König S, Bringmann H (2016). Sleep-active neuron specification and sleep induction require FLP-11 neuropeptides to systemically induce sleep. Elife.

[9] Kucherenko MM, Ilangovan V, Herzig B, Shcherbata HR, Bringmann H (2016). TfAP-2 is required for night sleep in Drosophila. BMC Neurosci.

[10] Moser M, Rüschoff J, Buettner R (1997). Comparative analysis of AP-2α and AP-2β gene expression during murine embryogenesis. Dev Dyn.

[11] Moser M, Dahmen S, Kluge R, Gröne H, Dahmen J, Kunz D (2003). Terminal renal failure in mice lacking Transcription factor AP-2β. Lab Investig.

[12] Nakai A, Fujiyama T, Nagata N, Kashiwagi M, Ikkyu A, Takagi M (2020). Sleep architecture in mice is shaped by the Transcription factor AP-2β. Genetics.

[13] Hu Y, Korovaichuk A, Astiz M, Schroeder H, Islam R, Barrenetxea J (2020). Functional divergence of mammalian TFAP2a and TFAP2b transcription factors for bidirectional sleep control. Genetics.

[14] Hu Y, Bringmann H (2023). Tfap2b acts in GABAergic neurons to control sleep in mice. Sci Rep.

[15] Hong SJ, Huh YH, Leung A, Choi HJ, Ding Y, Kang UJ (2011). Transcription factor AP-2β regulates the neurotransmitter phenotype and maturation of chromaffin cells. Mol Cell Neurosci.

[16] Zainolabidin N, Kamath SP, Thanawalla AR, Chen AI (2017). Distinct activities of Tfap2A and Tfap2B in the specification of GABAergic interneurons in the developing cerebellum. Front Mol Neurosci.

[17] Isaka F, Ishibashi M, Taki W, Hashimoto N, Nakanishi S, Kageyama R (1999). Ectopic expression of the bHLH gene Math1 disturbs neural development. Eur J Neurosci.

[18] Iwasaki K, Fujiyama T, Nakata S, Park M, Miyoshi C, Hotta-Hirashima N (2021). Induction of mutant Sik3 sleepy allele in neurons in late infancy increases sleep need. J Neurosci.

[19] Declercq J, Brouwers B, Pruniau VPEG, Stijnen P, de Faudeur G, Tuand K (2015). Metabolic and behavioural phenotypes in Nestin-Cre mice are caused by hypothalamic expression of human growth hormone. PLoS ONE.

[20] Zhang Z, Liu W-Y, Diao Y-P, Xu W, Zhong Y-H, Zhang J-Y (2019). Superior colliculus GABAergic neurons are essential for acute dark induction of wakefulness in mice. Curr Biol.

[21] Yao Y, Barger Z, Saffari Doost M, Tso CF, Darmohray D, Silverman D (2022). Cardiovascular baroreflex circuit moonlights in sleep control. Neuron.

[22] Hong SJ, Lardaro T, Oh MS, Huh Y, Ding Y, Kang UJ (2008). Regulation of the noradrenaline neurotransmitter phenotype by the Transcription Factor AP-2β. J Biol Chem.

[23] Yamaguchi H, Hopf FW, Li S-B, de Lecea L (2018). In vivo cell type-specific CRISPR knockdown of dopamine beta hydroxylase reduces locus coeruleus evoked wakefulness. Nat Commun.

[24] Pogorelov VM, Rodriguiz RM, Insco ML, Caron MG, Wetsel WC (2005). Novelty seeking and stereotypic activation of behavior in mice with disruption of the Dat1 gene. Neuropsychopharmacology.

[25] Won H, Lee H-R, Gee HY, Mah W, Kim J-I, Lee J (2012). Autistic-like social behaviour in Shank2-mutant mice improved by restoring NMDA receptor function. Nature.

[26] Silverman JL, Yang M, Lord C, Crawley JN (2010). Behavioural phenotyping assays for mouse models of autism. Nat Rev Neurosci.

[27] Berson DM, Dunn FA, Takao M (2002). Phototransduction by retinal ganglion cells that set the circadian clock. Science.

[28] Bassett EA, Korol A, Deschamps PA, Buettner R, Wallace VA, Williams T (2012). Overlapping expression patterns and redundant roles for AP-2 transcription factors in the developing mammalian retina. Dev Dyn.

[29] Jin K, Jiang H, Xiao D, Zou M, Zhu J, Xiang M (2015). Tfap2a and 2b act downstream of Ptf1a to promote amacrine cell differentiation during retinogenesis. Mol Brain.

[30] Kanki H, Suzuki H, Itohara S (2006). High-efficiency CAG-FLPe deleter mice in C57BL/6J background. Exp Anim.

[31] Liu J, Krautzberger AM, Sui SH, Hofmann OM, Chen Y, Baetscher M (2014). Cell-specific translational profiling in acute kidney injury. J Clin Invest.

[32] Hayashi Y, Kashiwagi M, Yasuda K, Ando R, Kanuka M, Sakai K (2015). Cells of a common developmental origin regulate REM/non-REM sleep and wakefulness in mice. Science.

