# Supplementary material for: Crucial role of TFAP2B in the nervous system for regulating NREM sleep
Source: Mol Brain. 2024 Feb 27;17:13. doi: 10.1186/s13041-024-01084-8 (PMC10900699; doi:10.1186/s13041-024-01084-8)
Supplement: Supplementary file 1 — Additional file 1. Fig. S1. Confirmation of Tfap2b deletion and comparison of bodyweight in nervous system-specific Tfap2b cKD and control mice. Fig. S2. Locomotor activity of nervous system-specific Tfap2b cKD mice. Fig. S3. Comparison of the amount of sleep/wake in nervous system-specific Tfap2b cKD and control mice during the active and inactive phase determined by locomotor activity. Fig. S4. Comparison of EEG power spectra in nervous system-specific Tfap2b cKD and control mice. Fig. S5. Confirmation of Tfap2b deletion and comparison of bodyweight in postnatal neuron-specific Tfap2b cKD and control mice. Fig. S6. Comparison of EEG power spectra in postnatal neuron-specific Tfap2b cKD and control mice. Fig. S7. Comparison of the sleep architecture after sleep deprivation (SD) in postnatal neuron-specific Tfap2b cKD and control mice. Fig. S8. Analyses of the Cre-mediated recombination pattern in Syn1CreERT2 mice at the LC. [file 13041_2024_1084_MOESM1_ESM.docx]

**Fig. S1. Confirmation of *Tfap2b* deletion and comparison of bodyweight in nervous system-specific *Tfap2b* cKD and control mice.**

(A) Results of PCR using genomic DNA obtained from the indicated tissues of WT mice, *Tfap2b^flox/flox^* mice, *Nes-Cre; Tfap2b^flox/+^* mice, and *Nes-Cre; Tfap2b^flox/flox^* mice. (B) Comparison of bodyweight in nervous system-specific *Tfap2b* cKD and control mice. Data on male mice are shown. *N*=13 *Tfap2b^flox/+^* mice (light grey), *N*=19 *Tfap2b^flox/flox^* mice (dark grey), *N*=15 *Nes-Cre; Tfap2b^flox/+^* mice (blue), *N*=8 *Nes-Cre; Tfap2b^flox/flox^* mice (pink). # and ‡indicate a significant main effect of genotype and significant interaction between genotype and weeks after birth, respectively, in 2-way repeated-measures ANOVA (####, ‡‡‡‡ *p*<0.0001). * indicates significance in post-hoc Bonferroni multiple comparison test (* *p*<0.05, ** *p*<0.01). Data are presented as mean ± SEM.

**Fig. S2. Locomotor activity of nervous system-specific *Tfap2b* cKD mice.**

(A-C) Patterns of infrared light-detected activity (5 min bin) across 2 consecutive days in individual *Tfap2b^flox/flox^* mice (A), *Nes-Cre; Tfap2b^flox/+^* mice (B), and *Nes-Cre; Tfap2b^flox/flox^* mice (C).

**Fig. S3. Comparison of the amount of sleep/wake in nervous system-specific *Tfap2b* cKD and control mice during the active and inactive phase determined by locomotor activity.**

Total amount of wakefulness, NREM sleep, and REM sleep in male mice during the inactive phase and the active phase, determined based on locomotor activity (see **Materials and methods** for details). *N*=9 *Tfap2b^flox/flox^* mice (grey), *N*=9 *Nes-Cre; Tfap2b^flox/+^* mice (blue), *N*=5 *Nes-Cre; Tfap2b^flox/flox^* mice (pink). # indicates a significant main effect of genotype in 2-way repeated-measures ANOVA (### *p*<0.001, #### *p*<0.0001). Data are presented as mean ± SEM.

**Fig. S4. Comparison of EEG power spectra in nervous system-specific *Tfap2b* cKD and control mice.**

Comparison of the EEG power spectrum of wakefulness, NREM sleep, and REM sleep for 24 h between *Tfap2b^flox/flox^, Nes-Cre; Tfap2b^flox/+^,* and *Nes-Cre; Tfap2b^flox/flox^* male mice. *N*=8 *Tfap2b^flox/flox^* mice (grey), *N*=9 *Nes-Cre; Tfap2b^flox/+^* mice (blue), *N*=6 *Nes-Cre; Tfap2b^flox/flox^* mice (pink). # and ‡ indicate a significant main effect of genotype and significant interaction between frequency and genotype, respectively, in 2-way repeated-measures ANOVA (##, ‡‡ *p*<0.01, ‡‡‡‡ *p*<0.0001). * indicates significance in post-hoc Bonferroni multiple comparison test (* *p*<0.05, ** *p*<0.01, *** *p*<0.001). Data are presented as mean ± SEM.

**Fig. S5. Confirmation of *Tfap2b* deletion and comparison of bodyweight in postnatal neuron-specific *Tfap2b* cKD and control mice.**

(A) Results of PCR using genomic DNA obtained from the indicated tissues of *Tfap2b^flox/flox^* mice and *Syn1^CreERT2^; Tfap2b^flox/flox^* mice injected with tamoxifen (at P14, 17, and 21). (B) Comparison of bodyweight. Data of tamoxifen-injected (at P14, 17, and 21) male mice are shown. *N*=9 *Tfap2b^flox/flox^* mice (grey), *N*=16 *Syn1^CreERT2^; Tfap2b^flox/flox^* mice (orange). Data are presented as mean ± SEM.

**Fig. S6. Comparison of EEG power spectra in postnatal neuron-specific *Tfap2b* cKD and control mice.**

Comparison of the EEG power spectrum of wakefulness, NREM sleep, and REM sleep for 24 h between *Tfap2b^flox/flox^* and *Syn1^CreERT2^; Tfap2b^flox/flox^* mice. Data of tamoxifen-injected (at P14, 17, and 21) male mice are shown. *N*=6 *Tfap2b^flox/flox^* mice (grey), *N*=8 *Syn1^CreERT2^; Tfap2b^flox/flox^* mice (orange). Data are presented as mean ± SEM.

**Fig. S7. Comparison of the sleep architecture after sleep deprivation (SD) in postnatal neuron-specific *Tfap2b* cKD and control mice.**(A) Hourly amount of wakefulness, NREM sleep, and REM sleep during 5 h of SD and subsequent 7 h in tamoxifen-injected (at P14, 17, and 21) male mice. For comparison, basal sleep data (same data as Fig. 2) are also shown. *N*=6 *Tfap2b^flox/flox^* mice (basal sleep; light grey), *N*=4 *Tfap2b^flox/flox^* mice (SD; dark grey). *N*=8 *Syn1^CreERT2^; Tfap2b^flox/flox^* mice (basal sleep; light orange), *N*=7 *Syn1^CreERT2^; Tfap2b^flox/flox^* mice (SD; dark orange). (B-D) Total amount (B), episode numbers (C), and durations (D) of wakefulness, NREM sleep, and REM sleep during the 7 h after SD (light phase). For comparison, basal sleep data (same data as Fig. 2) are also shown. *N*=6 *Tfap2b^flox/flox^* mice (basal sleep), *N*=4 *Tfap2b^flox/flox^* mice (SD). *N*=8 *Syn1^CreERT2^; Tfap2b^flox/flox^* mice (basal sleep), *N*=7 *Syn1^CreERT2^; Tfap2b^flox/flox^* mice (SD). (E) Comparison of the EEG power spectrum of wakefulness, NREM sleep, and REM sleep for 7 h after SD (light phase). *N*=4 *Tfap2b^flox/flox^* mice (grey), *N*=7 *Syn1^CreERT2^; Tfap2b^flox/flox^* mice (orange). $ and # indicate a significant main effect of SD and genotype, respectively, in 2-way repeated-measures ANOVA ($, # *p*<0.05, $$, ## *p*<0.01, $$$ *p*<0.001, $$$$ *p*<0.0001). Data are presented as mean ± SEM.

**Fig. S8. Analyses of the Cre-mediated recombination pattern in *Syn1^CreERT2^* mice at the LC.**

(A, B) Images of areas including the LC in brain slices that underwent immunostaining for tyrosine hydroxylase (TH) derived from *Rosa26^LSL-L10-GFP/+^* (control; A) and *Syn1^CreERT2^;* *Rosa26^LSL-L10-GFP/+^* (B) mice. Scale bars; 100 µm. Blue; DAPI. Magenta; TH. Green; GFP. Representative images from a replicate of *N* =2 *Rosa26^LSL-L10-GFP/+^* mice, *N* =3 *Syn1^CreERT2^;* *Rosa26^LSL-L10-GFP/+^* mice are shown.

**Video1. Representative video of nervous system-specific *Tfap2b* cKD and control mice.**(A-D) Video recordings of an individual *Nes-Cre; Tfap2b^flox/+^* (A), *Tfap2b^flox/flox^* (B), *Nes-Cre; Tfap2b^flox/+^* (C), or *Nes-Cre; Tfap2b^flox/flox^* (D) male mouse during the light phase.

**Video2. Representative video of postnatal neuron-specific *Tfap2b* cKD and control mice.**(A, B) Video recordings of an individual *Syn1^CreERT2^; Tfap2b^flox/flox^* (A) or *Tfap2b^flox/flox^* (B) male mouse injected with tamoxifen (at P14, 17, and 21) during the light phase.

# Materials and methods

## Mice

All animal experiments were approved by the institutional animal care and use committee of the University of Tsukuba. All animals were maintained according to the institutional guidelines of the animal facilities of the Laboratory of Animal Resource Center, University of Tsukuba. Mice were maintained and bred within the International Institute for Integrative Sleep Medicine under a 12-h light/dark cycle and with free access to food and water. Male *Nes-Cre; Tfap2b^flox/flox^*, *Syn1^CreERT2^; Tfap2b^flox/flox^*, and corresponding control mice aged 10–13 weeks were used for sleep analyses. To obtain *Tfap2b* cKD mice, *Tfap2b^tm1a(EUCOMM)Wtsi/+^* mice were crossed with mice carrying the *CAG-flp* allele [30] to remove a genomic DNA fragment containing IRES:lacZ and a neomycin-resistant cassette flanked by FRT. Homozygous floxed *Tfap2b* exon 3 (*Tfap2b^flox/flox^*) mice were mated to mice carrying the *Nes-Cre* transgene [17] or the *Syn1^CreERT2^* allele [18] to induce Cre-mediated recombination in a nervous system-specific manner or a neuron-specific manner, respectively.

To confirm the recombination sites in the *Nes-Cre* and *Syn1^CreERT2^* mice, *Nes-Cre* and *Syn1^CreERT2^* mice were mated with the GFP reporter strain *B6.129S4-Gt (ROSA)26Sor^tm1(CAG-EGFP/Rpl10a-birA) Wtp/J^* (*Rosa26^LSL-L10-GFP^*, Jackson Laboratory ; #022367) [31].

To induce CreERT2-mediated recombination, 500 μg of tamoxifen (Toronto Research Chemicals Inc.; T006000) dissolved in corn oil was administered intraperitoneally at P14, P17, and P21.

## Genotyping

Genotyping of mice carrying *Tfap2b^flox^*, *Nes-Cre*, *Syn1^CreERT2^*, or *Rosa26^LSL-L10-GFP^* was performed using the following primers. *Tfap2b^flox^*: 5′-AAGGCGCATAACGATACCAC-3′, 5′-CCGCCTACTGCGACTATAGAGA-3′, 5′-GACATCCTACAATGCACAGCT-3′, and 5′- TTGCTGTGAGCTAAGAGCTTC-3′ (*Tfap2b^flox^*: 218 bp, *Tfap2b^+^*: 381 bp), *Nes-Cre*: 5′-GACGATGCAACGAGTGATGA-3′ and 5′-AGCATTGCTGTCACTTGGTC-3′ (*Nes-Cre*: 300 bp), *Syn1^CreERT2^*: 5′-TGCCTCCACCTTGTCTCTCT-3′, 5′-AACAAAGGCATGGAGCATCT-3′, and 5′-GATCTGGAGGTGACCAGGAA-3′ (*Syn1^CreERT2^*: 443 bp, *Syn1^+^*: 387 bp), *Rosa26^LSL-L10-GFP^*: 5′-AAGGGAGCTGCAGTGGAGTA-3′, 5′-CCGAAAATCTGTGGGAAGTC-3′, 5′-AAGATCCGCCACAACATCG-3′, and 5′-TTCTCGTTGGGGTCTTTGCT-3′ (*Rosa26^LSL-L10-GFP^*: 146 bp, *Rosa26^+^*: 297 bp).

## PCR using genomic DNA extracted from brain Each brain was harvested and divided in half at the midline and frozen with liquid nitrogen. Genomic DNA was extracted using ISOSPIN Tissue DNA Kit (NIPPON GENE; 316-08891). PCR was performed using the following primers. *Tfap2b^-^*: 5′-AAGGCGCATAACGATACCAC-3′ and 5′-ACTGATGGCGAGCTCAGACC-3′ (*Tfap2b^-^*: 174 bp, *Tfap2b^flox^*: 1024 bp), *Chn1*: 5′-AGGGCTTTCCTTGCTGTGTC-3′ and 5′-TAGGTCCCTTCTCATGAACC-3′ (*Chn1^+^*: 120 bp).

## EEG/ electromyogram (EMG) and locomotor recording

EEG and EMG signals were recorded from freely moving mice at the age of 10–13 weeks according to the method described in a previous study [32], with some modifications. First, 8–10 weeks-old mice were implanted with EEG/EMG electrodes. Stainless steel EEG electrodes were implanted epidurally over the cerebellum and parietal cortex, and EMG electrodes were embedded into the trapezius muscles bilaterally. Mice were allowed to recover from the surgery in their home cage for at least 4 days. Then, mice were placed in a sleep recording chamber and habituated for at least 5 days. Subsequently, EEG/EMG signals were recorded from the onset of the light phase. EEG/EMG signals were filtered (bandpass 0.5–250 Hz), collected, and digitized at a 512 Hz sampling rate using VitalRecorder (Kissei Comtec). Video and infrared signals were also recorded from the start of the light phase. Locomotor activity detected by infrared signals was used to determine the onset of the active phase, which was automatically done using a template-matching algorithm in the ClockLab software. Briefly, the onset of the active phase was defined as the start point of 5 consecutive hours of high locomotor activity (above the 20% percentile of average locomotor activity) that followed 5 consecutive hours of low locomotor activity. 12 consecutive hours from the onset of the active phase were categorized as the active phase, and the remaining 12 consecutive hours were categorized as the inactive phase.

## EEG/EMG analysis

EEG/EMG data were divided into 4-s epochs (time window), and EEG data were further subjected to fast Fourier transform analyses using SleepSign (Kissei Comtec). The vigilance state in each epoch was manually classified as REM sleep, NREM sleep, or wake based on absolute delta (0.5–4 Hz) power, theta (6–10 Hz) power to delta power ratio, and the integral of EMG signals. If a single epoch contained multiple states, the state with the highest occupancy was assigned. The EEG power spectrum of each state was calculated and normalized by EEG power at 16–30 Hz averaged across 24 h. Epochs that contained multiple stages or presumable large movement-derived artifacts in the EEG data were included in the stage analysis but excluded from the EEG power spectrum analysis.

## Sleep deprivation (SD)

SD was performed for 5 h during ZT0-ZT5. We monitored EEG/EMG to detect sleep. The cage was changed at the start of SD. Whenever sleep was detected, Kimwipes or marbles were placed in or removed from the cage, or the cage was gently tapped.

## Histology

Deeply anesthetized mice were killed by injecting a lethal dose of anesthetic and transcardially perfused with 0.1 M phosphate-buffered saline (PBS) followed by 4% paraformaldehyde (w/v) in 0.1 M PBS or 10% formalin neutral buffer solution. The brains were postfixed in the same fixative for 1 day and subsequently equilibrated in 30% sucrose (w/v) in PBS. The brains were sectioned at 40 μm using a sliding microtome (Yamato Kohki) or a cryostat (Leica).

## Immunohistochemistry

Sections were washed 3 times in tris-buffered saline with Tween20 (TBST; 50 mM Tris-HCl, pH 7.6, 150 mM NaCl, 0.05% Tween20), once in 0.3% H_2_O_2_/TBST, and 3 times again in TBST. The sections were then blocked for 30 min in blocking buffer (tris-buffered saline with 0.5% Blocking Reagent (Perkin Elmer; FP1012)). The sections were incubated with a primary antibody (1/2000 mouse anti-tyrosine hydroxylase (Sigma; T2928)). After washing 3 times in TBST, the sections were incubated with a secondary antibody (1/500 horseradish peroxidase-conjugated donkey anti-mouse IgG (Abcam; ab7061)) for 2 h. After washing 3 times in TBST, the sections were incubated with Tyramide Signal Amplification (TSA) plus Cyanine 5 reagent (Perkin Elmer; NEL745001KT) for 30 min. Finally, after washing 4 times in TBST, the sections were mounted on a slide glass with mounting medium (ThermoFisher; TA-006-FM). Images were captured with a digital slide scanner (Carl Zeiss; AxioScan.Z1) or a confocal microscope (Carl Zeiss; LMS800).

## Quantification and statistical analysis

The experimenter was blinded to the genotype during sleep scoring. All statistical analyses were performed using Prism9 (GraphPad), and statistical significance was set at *p* < 0.05. Where applicable, all statistical tests were 2-tailed.
